# The Role of Endoplasmic Reticulum Stress in Cardiovascular Disease and Exercise

**DOI:** 10.1155/2017/2049217

**Published:** 2017-08-10

**Authors:** Junyoung Hong, Kwangchan Kim, Jong-Hee Kim, Yoonjung Park

**Affiliations:** ^1^Laboratory of Integrated Physiology, Department of Health and Human Performance, University of Houston, Texas, TX 77204, USA; ^2^Department of Physical Education, Hanyang University, Seoul, Republic of Korea

## Abstract

Endoplasmic reticulum (ER) stress, which is highly associated with cardiovascular disease, is triggered by a disturbance in ER function because of protein misfolding or an increase in protein secretion. Prolonged disruption of ER causes ER stress and activation of the unfolded protein response (UPR) and leads to various diseases. Eukaryotic cells respond to ER stress via three major sensors that are bound to the ER membrane: activating transcription factor 6 (ATF6), inositol-requiring protein 1*α* (IRE1*α*), and protein kinase RNA-like ER kinase (PERK). Chronic activation of ER stress causes damage in endothelial cells (EC) via apoptosis, inflammation, and oxidative stress signaling pathways. The alleviation of ER stress has recently been accepted as a potential therapeutic target to treat cardiovascular diseases such as heart failure, hypertension, and atherosclerosis. Exercise training is an effective nonpharmacological approach for preventing and alleviating cardiovascular disease. We here review the recent viewing of ER stress-mediated apoptosis and inflammation signaling pathways in cardiovascular disease and the role of exercise in ER stress-associated diseases.

## 1. Introduction

The endoplasmic reticulum (ER) plays an essential role in controlling various intracellular physiological functions, including protein translocation, protein folding, calcium homeostasis, and lipid biosynthesis [1], by stimulating the signaling networks that control either cell survival or death when ER transmembrane sensors detect unfolded and misfolded proteins. Various pathophysiological conditions disturb ER homeostasis and can lead to the chronic activation of the unfolded protein response (UPR) in ER lumen, which causes ER stress. Prolonged ER stress activates three ER transmembrane sensors and initiates UPR signaling, which induces oxidative stress, inflammation, and apoptotic response. It aggravates neurodegenerative disease, cancer, metabolic disease, and kidney disease [2, 3] and is highly associated with cardiovascular diseases such as cardiac hypertrophy, heart failure, atherosclerosis, and ischemic heart disease [4–7]. In particular, ER stress-induced endothelial cell (EC) damage and dysfunction exert the negative influences on cardiovascular diseases through apoptosis and inflammation [8].

It has been well established that exercise training can improve EC function and decrease the risk of cardiovascular diseases by increasing nitric oxide (NO) bioavailability, diminishing the volume of plaque and vascular viscosity, and increasing both diastolic coronary perfusion and vascular reactivity [9, 10]. Numerous studies report that exercise could effectively improve various ER stress-related pathologies such as obesity, diabetes, neurodegenerative disease, hypoxia, and sarcopenia in skeletal muscle, liver, brain, and cardiovascular systems [11–17]. In this review, we focus on the mechanism of ER stress-mediated apoptosis and inflammation in cardiovascular disease, and the effect of exercise on ER stress-associated diseases.

## 2. ER Stress and UPR Signaling

The ER is an intracellular organelle covered by an extensive membrane network and is present in all eukaryotic cells. It is the major site of protein synthesis, protein folding, protein transport, lipid production, and calcium storage [18]. Multiple biological insults such as calcium and redox imbalance, viral infection, oxidative stress, and hyperlipidemia lead to perturbation of ER homeostasis, which consequently initiates the accumulation of unfolded proteins in the ER lumen that is called ER stress [19, 20].

**Figure 1:**
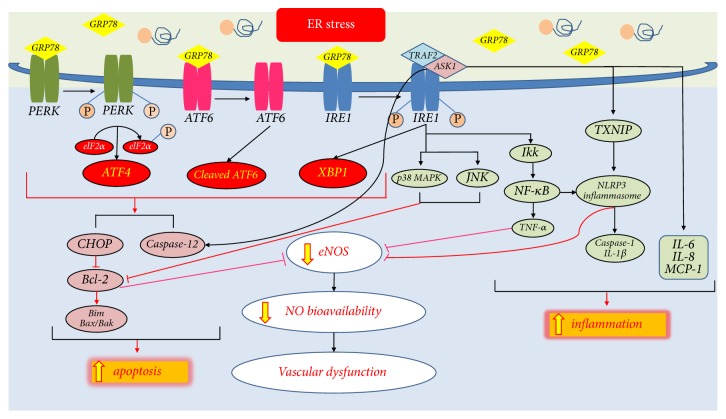
*ER stress-mediated apoptosis and inflammation in cardiovascular disease*. When prolonged ER stress occurs, activation of CHOP-mediated and JNK/p38 MAPK-mediated apoptosis signaling pathways regulate the Bcl-2 family, which controls the balance of pro-/antiapoptotic signaling, and ER stress-induced apoptosis signaling dampens eNOS expression with the increased oxidative stress. The IRE1*α*/TRAF2/ASK1 complex also activates caspase-12 and ultimately induces cell apoptosis. Furthermore, ER stress-mediated NF-*κ*B-IKK activates NLRP3 inflammasome and mediates eNOS activity. Excessive ER stress and activation of the IRE1*α*/TRAF2/ASK1 pathway increase TXNIP activation that subsequently induces NLRP3 inflammasome and reduced eNOS activity. The IRE1*α*/TRAF2/ASK1 complex also contributes to the expression of proinflammations that stimulate NLRP3 inflammasome-induced caspase-1 and IL-1*β* and UPR-mediated IL-6. ER stress also activates IL-8, MCP-1, and TNF-*α* expression. Ultimately, the accumulation of ER stress-mediated apoptotic and inflammatory responses decreases the eNOS activity which diminishes NO bioavailability and causes vascular dysfunction. The yellow arrows represent ER stress-mediated pathway.

**Figure 2:**
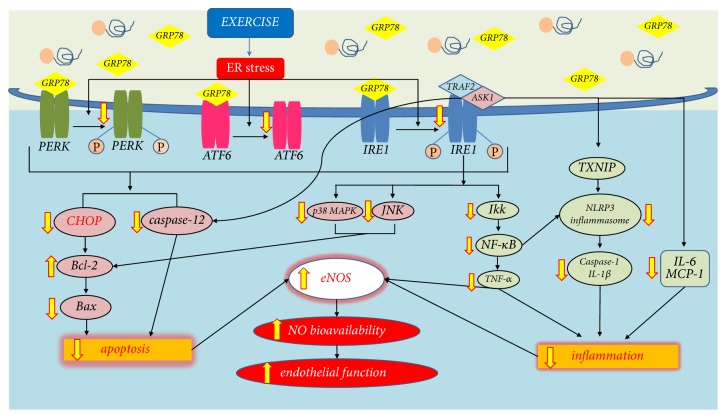
*The Chronic effect of exercise on the ER stress-associated apoptosis and inflammation.* Regular exercise reduces phosphorylation of the three ER stress sensors and inhibits or decreases ER stress-associated apoptosis and inflammation signaling pathways. Exercise training ameliorates ER stress-mediated CHOP signaling, in turn reducing apoptosis. Exercise training also reduces IRE1*α*-mediated p38 MAPK/JNK and NF-*κ*B-associated inflammasome regulating the expression of caspase-1, IL-1*β*, IL-6, and MCP-1, mitigates the IRE1*α*/TRAF2/ASK1 complex that links to caspase-12, and mediates apoptosis in cardiovascular disease. As a result, these signaling pathways systemically mediate eNOS expression through the decreased apoptosis and inflammation, which increase NO bioavailability and improve endothelial function. The yellow arrows represent the exercise-induced changes in ER stress-mediated apoptosis and inflammatory pathway.

The UPR, which is chiefly responsible for determining cellular death or survival responses in accordance with ER stress, is designed to restore ER homeostasis by activating ER-associated protein degradation (ERAD), autophagy, and cell survival signals [21]. Excessive ER stress, however, activates UPR and promotes ER stress-associated signaling cascades that stimulate apoptosis and inflammatory signaling pathway [22, 23] (Figure 1). The UPR generally senses the misfolded proteins in the ER and activates each signaling pathway through three sensors bound to the ER membrane (Figure 2): PKR-like eukaryotic initiation factor 2*α* kinase (PERK), inositol-requiring protein 1 (IRE1), and activating transcription factor-6 (ATF6) [24, 25]. In unstressed conditions, the transmembrane proteins bind to glucose-regulated protein 78 (GRP78 or BiP), a molecular chaperone that inhibits the activities of these transmembrane proteins [25]. When ER stress occurs, GRP78 dissociates from the three sensors and initiates the UPR. By the onset of ER stress, the PERK signaling pathway decreases protein synthesis by reducing protein load in ER lumen and the activated PERK phosphorylates eukaryotic initiation factor-*α* (elF2*α*). Eventually, the process induces cell death via certain gene families including activating transcription factor-4 (ATF4) and C/EBP homologous protein (CHOP) [1]. IRE1 promotes the activation of major inflammatory response factors in signaling pathways and induces splicing of a transcriptional regulatory factor in the bZip family, called X-box binding protein-1 (XBP-1), through its site-specific endoribonuclease (RNase) function [26]. When ATF6 is released by BiP, it is translocated and activated after proteolysis in the Golgi apparatus [27]. In turn, ATF6 activates the synthesis of ER chaperones and XBP-1. Therefore, the three branches of UPR signaling (ATF4, XBP-1, and ATF6) are highly activated to regulate the transcription of ER chaperones in ER stress that blocks protein synthesis and decreases protein folding capacity [28, 29].

## 3. ER Stress-Mediated Apoptosis in Cardiovascular Disease

ER stress has been highlighted as an important regulator of cardiovascular diseases [30–32]. The endothelium is the crucial site to maintain vascular homeostasis and control vascular reactivity via endothelium-derived relaxing factors (EDRFs) [33]. Endothelial dysfunction is the initial response in many cardiovascular and metabolic diseases. CHOP, the most widely investigated biomarker involved in ER stress-associated apoptotic signaling in cardiovascular disease, is regulated by anti- and proapoptotic protein of Bcl-2 family [32]. In atherosclerosis, the UPR fails to control misfolded proteins in the ER and increases the expression of CHOP with a progression of atherosclerosis in the aorta. Eventually, it activates CHOP-induced apoptosis signaling and further responses [6, 30–32, 34]. PERK/ATF4 and ATF6-dependent pathways regulate the CHOP-mediated proapoptotic bZIP transcriptional factor and IRE1-dependent apoptotic signaling is activated through various processes [34, 35]. IRE1 interacts with the TNF receptor-associated factor (TRAF) 2; in turn, the complex of IRE1 and TRAF2 are associated with apoptosis signal-regulating kinase 1 (ASK1), which activates both the c-Jun N-terminal kinase (JNK) and p38 mitogen-activated protein kinases (MAPK) [35, 36].

The Bcl-2 family of genes is an important apoptotic factor for controlling the balance of proapoptotic and antiapoptotic signals when both CHOP and IRE1 are activated [37] (Figure 2). The Bcl-2 family includes both antiapoptotic and proapoptotic members that mediate the crosstalk between the ER and mitochondria. Bax and Bak are the most well-known proapoptotic members and Bcl-2 and Bcl-x are the most widely known antiapoptotic members of the Bcl-2 family. Activating the proapoptotic proteins on the mitochondrial membrane releases cytochrome-c and causes subsequent apoptosis induction [38]. Because of the decline of mitochondrial function in ECs via the CHOP-mediated proapoptotic proteins [39] and the interrupted calcium homeostasis [40], the levels of reactive oxygen species (ROS) and nicotinamide adenine dinucleotide phosphate (NADPH) are increased in ECs in atherosclerosis [41, 42]. It is unclear whether NADPH oxidases act as upstream or downstream of ER stress-induced cardiovascular dysfunction. Recent studies report that ER stress-associated oxidative stress and inflammation suppress the production of NO and endothelial nitric oxide synthase (eNOS) activity, both of which protect ECs [8, 30, 43–45]. The ER stress-induced activation of JNK causes oxidative stress and vascular endothelial dysfunction through repression of eNOS activity [46, 47].

The caspase-12 protein regulates ER stress-induced apoptosis signaling [31, 48] (Figure 2). Two pathways activate procaspase-12. First, the elevated Ca^2+^ level in the cytoplasm activates procaspase-12 in the ER membrane to form caspase-12; the process requires calpain that induces activation of caspase-9 and finally activates caspase-3 and apoptosis [49]. Second, during ER stress, TRAF2 dissociates from the complex of TRAF2/procaspase-12 located in the ER membrane; the process activates caspase-12 and recruits the IRE1/JNK/TRAF2 complex that modulates ASK1 that in turn phosphorylates JNK and induces cell apoptosis [50]. Ischemia/reperfusion (I/R) showed the elevated level of caspase-12 and ER stress markers in myocardium [46, 51–53], and the expression of caspase-12 and cleaved caspase-3 were abnormally altered in the pathological cardiac hypertrophy in rodent model [54, 55].

## 4. ER Stress-Mediated Inflammation in Cardiovascular Diseases

Inflammation is an immunological response to infection or tissue damage and protects the body from such injuries. Chronic inflammation aggravates tissue damage, and it can influence the development of cardiac hypertrophy, heart failure, coronary artery diseases, and atherosclerosis [56, 57]. ER stress and inflammation signaling pathways are connected through various mechanisms that can induce cardiovascular disease (Figure 2). In atherosclerosis, increased PERK and IRE1/TRAF2 and accumulated ROS activate and augment inflammatory response [58]. PERK and IRE1/TRAF2 complex can recruit I*κ*B kinase (IKK), which phosphorylates I*κ*B, resulting in the degradation of I*κ*B and the nuclear translocation of NF-*κ*B [59]. The NF-*κ*B-IKK pathway is a key regulator in the induction of inflammatory mediators [59, 60]. Recent studies report that ATF6 also interacts with NF-*κ*B-IKK, indicating that all three sensors of ER stress (PERK, IRE1, and ATF6) can induce specific inflammatory responses through the UPR [61]. Induction of the UPR at the cellular level increases the expression of inflammatory molecules, including IL-8, IL-6, MCP-1, and TNF-*α*, thereby inducing atherosclerosis [62]. Other studies report that TNF-*α* and NF-*κ*B signaling through the IKK pathway can provoke coronary arteriolar dysfunction [63]. TNF-*α* reduces the bioavailability of NO by increasing the activity of NADPH and downregulating eNOS [63–66]. IL-1*β* and TNF-*α* are associated with the increased expression of vascular cell adhesion molecule 1 (VCAM-1) and intercellular cell adhesion molecule 1 (ICAM-1) [67]. IL-1*β* and TNF-*α* also stimulate apoptosis through promoting the expression of caspase family and inducible NOS (iNOS) in ECs [68–70].

Other recent studies report that NF-*κ*B, an immunological mediator, plays a vital role in controlling the NOD-like receptor family, pyrin domain containing 3 (NLRP3) inflammasome [71–73]. Notably, IRE1 induces an elevation of thioredoxin-interacting protein (TXNIP); the elevated TXNIP promotes inflammation and cell apoptosis by activating NLRP3 inflammasome that in turn activates caspase-1 to induce the secretion of IL-1*β* [73–75] (Figure 2). Activated caspase-1 is usually observed in ruptured plaques; the survival rate of patients who have high plasma levels of caspase-1 is much lower than the individuals with a normal level of plasma caspase-1 [76]. In addition, NLRP3 inflammasome-induced increases in IL-1*β* are known to upregulate proinflammatory and proapoptotic genes in ECs [77]. ER stress-associated TXNIP/NLRP3 signaling is activated in a high concentration of palmitate-treated endothelial cells and in the high fat diet-fed mice aorta. It induces the endothelial dysfunction through enhanced oxidative stress and reduced eNOS expression [78]. Pharmacological treatment using AMP-activated protein kinase (AMPK) can improve the endothelial dysfunction caused by the activation of ER stress-associated TXNIP/NLRP3; AMPK improves mitochondrial morphology and endothelial dysfunction by repressing mitochondrial ROS-associated ER stress-dependent activation of the TXNIP/NLRP3 inflammasome [79].

Perturbation of ER stress and UPR leads to a toxic intracellular accumulation of ROS, a possible cause of ER stress-associated inflammation [1, 80]. Alteration of PERK and ATF4 signaling is responsible for ER stress-associated redox imbalance and affects the disulfide bond formation (ERO1, endoplasmic reticulum oxidoreductin; PDI, protein disulfide isomerase) that influences antioxidant activation in ER [2]. A PERK-mediated Nrf2 cascade impairs the antioxidant process that stimulates I*κ*B/NF-*κ*B signaling, resulting in increased inflammatory response of IL-6 and TNF-*α* expression during ER stress [1, 60, 81].

## 5. ER Stress and Exercise

Regular exercise is considered an effective tool to prevent and reduce the risk of cardiovascular disease [33]. Endothelial dysfunction with the reduction of NO bioavailability is a common symptom of hypertension, obesity, heart failure, and atherosclerosis [64, 82–84]. Exercise training provides the numerous positive effects on endothelial dysfunction and helps maintain cardiovascular homeostasis through an increase in antioxidative response and a reduction of inflammatory cytokines expression [10, 56, 82, 85, 86]. The beneficial effect of exercise on ER stress, however, will depend on the modality and duration of exercise. There is still insufficient data to establish the definitive effects of exercise training on ER stress-associated cardiovascular disease. Therefore, this section discusses the overall exercise effects on ER stress-associated diseases.

**Figure 3:**
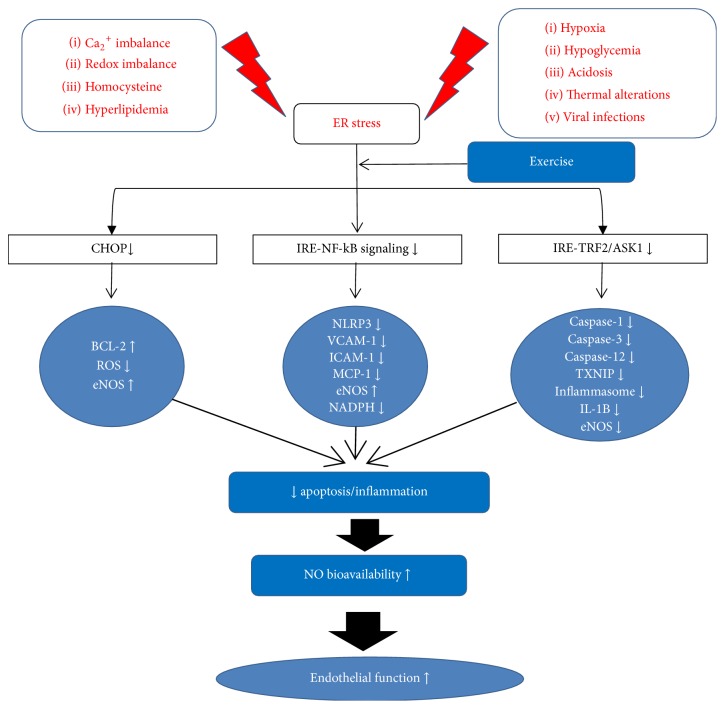
*Schematic diagram of the chronic effect of exercise on ER stress and endothelial function*. ER stress-mediated apoptosis and inflammatory responses are altered by exercise training; (1) CHOP-mediated, (2) IRE1*α*/NF-*κ*B complex-associated, and (3) IRE1*α*/TRAF2/ASK1-associated gene/protein expression of apoptosis and inflammation are downregulated and eventually increase endothelial function by the elevated NO bioavailability.

Chronic aerobic exercise training shows the diverse patterns in the expression of ER stress markers (Figure 3). Treadmill exercise can improve cardiac function and reduce cardiac infarction by attenuating the expression of GRP78, DERLIN-1, p-PERK, p-eIF2*α*, ATF4/6, XBP1, CHOP, and cleaved caspase-3 [11, 87]. Treadmill exercise has been shown to ameliorate ER stress (p-eIF2*α*, ATF3, and ATF4) and endothelial dysfunction (conduit and resistance vessels) in diabetic mice through a PPAR*γ*-dependent mechanism with an increase in NO bioavailability [88]. The expressions of ER stress markers (GRP78, p-PERK, p-elF2*α*, p-IRE1*α*, p-ATF6*α*, p-sXBP1, and p-CHOP), ER stress-induced apoptotic proteins (procaspase-3, 12, p-JNK, Bax, and Bcl-2), and ER stress-induced inflammatory cytokines (NLRP3/IL-1*β* and proinflammatory cytokines) were decreased after treadmill exercise in an obese rodent [12, 13, 15]. Swimming exercise reduced p-PERK, p-eIF2*α*, JNK, IkB*α*, and NF-*κ*B in an obese mice model [13, 15, 89]. However, one study has reported that treadmill exercise training elevated UPR response (PERK, IRE1*α*) with a reduction of inflammation (IL-6 and MCP-1) in high fat diet-induced mice model [90]. After 3 months of combination of aerobic and resistance exercises training, mRNA and protein levels of GRP78, p-IRE1*α*, and p-eIF2*α* were decreased in subcutaneous adipose tissue and peripheral blood mononuclear cells (PBMCs) in obese adult subjects [16].

After mice completed 8 weeks of uphill and downhill running, downhill running increased the BiP, ATF6, p-IRE1-*α*, and p-PERK expression in the extensor digitorum longus (EDL) muscle compared with uphill running. The finding suggests that eccentric contraction-induced muscle injuries stimulated the ER stress [91]. Furthermore, GRP78 was significantly elevated in both low and high intensity acute aerobic exercise training in rat model and adolescents with type 2 diabetic patients [92, 93]. ER stress-related gene and protein expression (p-PERK, XBP-1s, p-eIF-2*α*), respectively, increased but no changes of apoptosis signaling were found in high fat diet-induced obese and muscular dystrophy mice models after 3 or 7 weeks of voluntary exercise [14, 94]

Short-term aerobic exercise is not sufficient to induce ER stress adaptation in animal models. GRP78, CHOP, cleaved/procaspase-12, and ATF3 were not altered after 5 days of aerobic training in rat model, and ER stress-related protein expression was not affected by one-day swimming exercise in TLR4-deficient mice [95, 96]. Acute exercise changes the expression of UPR/ER stress signaling. CHOP-mediated apoptosis signaling (Bax, Bcl-2, and caspase-3, 12) was activated with the elevated intracellular Ca^2+^ after rats completed one bout of swimming exercise [96]. In contrast, acute resistance exercise by untrained men elevated UPR signaling but did not change CHOP expression [97].

Altogether, exercise training has been shown to mitigate all three ER stress markers, ER stress-mediated inflammation, and apoptosis in metabolic and chronic disease model. However, published ER stress-associated studies on skeletal muscle showed that ER stress markers such as GRP78, p-PERK, p-IRE1, and CHOP were activated and elevated after different types of aerobic and resistance training [91, 97–99]. Interestingly, additional studies reported that the mice group which had previous training showed the decreased activation of UPR and CHOP genes compared to untrained mice after equal amount of treadmill exercise [98]. Also, the elevated mRNA levels of UPR genes (Bip, ATF4, and XBP1s) and CHOP, as well as CHOP protein expression after initial response to chronic contractile activity (CCA), were attenuated with repeated bouts of CCA in rats [100].

Therefore, these study results suggested that initial response to exercise training induces ER stress and activate UPR signaling. However, after prolonged exercise training, these increased markers are alleviated, which suggested that the activated UPR signaling is induced to acclimate to exercise training for cell survival and adaptation.

## 6. Conclusion

ER, an essential organelle for cell homeostasis, plays a central role in cell death and survival signaling. While myriad studies have demonstrated that chronic ER stress is one of the major contributors to cardiovascular disease, it is not precisely understood how ER stress modulates endothelium-dependent vascular function, especially NO signaling pathway. Exercise training elicits a beneficial effect on the endothelial function by increasing the antioxidative/inflammatory response in cardiovascular system. Additional well-designed, controlled studies of the exercise effect on ER stress-mediated cardiovascular disease and the mechanisms that trigger ER stress-associated cardiovascular dysfunction will close the existing knowledge gap and lead to the development of new therapeutic targets.
